# Medium‐Term Follow‐Up Outcomes of One‐Stage Posterior Lumbosacral or Lumbopelvic Fixation in the Management of Lumbosacral Junction Tuberculosis in Adults

**DOI:** 10.1111/os.13150

**Published:** 2021-09-30

**Authors:** Zhenchao Xu, Zhen Zhang, Xiyang Wang, Yilu Zhang, Yunqi Wu

**Affiliations:** ^1^ Department of Spine Surgery and Orthopaedics The Xiangya Hospital of Central South University Changsha China; ^2^ Hunan Engineering Laboratory of Advanced Artificial Osteo‐Materials Changsha China

**Keywords:** Lumbosacral region, Orthopaedic surgery, Posterior approach, Spinal tuberculosis, Spondylodiscitis

## Abstract

**Objective:**

To evaluate the medium‐term outcomes of one‐stage posterior lumbosacral or lumbopelvic fixation treatment of lumbosacral junction tuberculosis in adults.

**Methods:**

This retrospective study enrolled a total of 38 adult patients (24 males and 14 females) with an average age of 48.0 ± 13.0 years (range, 25–75 years) during the period from February 2008 to July 2015. All patients were treated by one‐stage posterior debridement, interbody fusion, lumbosacral or lumbopelvic fixation, and postural drainage. After pedicle screw or iliac screw fixation, a hemi‐laminectomy or laminectomy was performed on the severely damaged side of the lesion segment. Intervertebral bone grafting and intertransverse bone grafting were performed after clearing the focus of tuberculosis. All cases were followed up for at least 5 years. Intraoperative blood loss, operative time, erythrocyte sedimentation rate (ESR), pain intensity was assessed by visual analog scale (VAS) score; neurological function was assessed by Japanese Orthopaedic Association (JOA) score; quality of life was assessed by Oswestry Disability Index (ODI); functional outcome, lumbosacral angle, and fusion time were gathered and analyzed. All data expressed as mean ± standard deviation.

**Results:**

During the 66.2 ± 4.4 months (range, 60–78 months) follow‐up, all patients achieved clinical cure without severe complications. The intraoperative blood loss was 726.3 ± 151.9 mL (range, 400–1100 mL) and the operative time was 137.6 ± 22.5 min (range, 110–200 min). The ESR decreased to normal levels within (11.8 ± 2.6 mm/h) 3 months postoperatively. The VAS score significantly decreased from 6.8 ± 1.1 preoperatively to 0.8 ± 0.7 at the final follow‐up (*P* < 0.01). The mean JOA improved from preoperative 18.5 ± 2.9 to 26.9 ± 1.1 at the last visit (*P* < 0.01). The mean ODI was 44.3 ± 6.7 and significantly decreased to 9.3 ± 1.9 at the final observation (*P* < 0.01). Patient‐reported outcomes as measured by Kirkaldy‐Willis criteria were excellent in 21 cases, good in 16 cases, and fair in one case; there were no poor outcomes. Lumbosacral angle increased from the preoperative values of 21.7° ± 1.8° to the postoperative values of 26.4° ± 1.4° (*P* < 0.01), with an angle loss of 1.2° ± 0.7° at the last follow‐up. Bone fusion occurred on average 12.8 ± 1.9 months (range, 9–15 months) after surgery. No nonunion, pseudarthrosis, loosening or fracture of instruments occurred at the last follow‐up.

**Conclusion:**

One‐stage posterior debridement, interbody fusion, lumbosacral or lumbopelvic fixation, and postural drainage according to the severity of sacral destruction is an effective and highly safe procedure to treat lumbosacral junction tuberculosis in adults.

## Graphical abstract

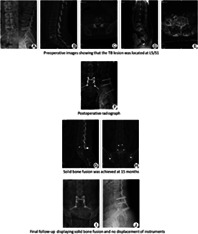

We evaluated the medium‐term outcomes of one‐stage posterior lumbosacral or lumbopelvic fixation treatment of lumbosacral junction tuberculosis in adults. This surgical approach is an effective and highly safe procedure, which can lead to reconstruction of lumbosacral or lumbopelvic stability, while facilitating healing from TB‐related inflammation.

## Introduction

According to the Global Tuberculosis Report released by the World Health Organization in 2019, there were approximately 10 mn new cases of tuberculosis (TB) worldwide in 2018, making it one of the top 10 causes of death in the world^1^. The spinal column is the most common site of infection for extrapulmonary TB, accounting for approximately 50% of osteoarticular TB^2^. This severe spinal disease often causes spinal instability, kyphotic deformity, neurological dysfunction, and even paraplegia^3^. Spinal TB involving the lumbosacral junction is rare, and only accounts for 2%–3% of the total number of spinal TB cases reported in the literature^4^. Conservative treatment with anti‐TB drugs is the primary approach for the condition^5^. However, simple drug therapy is often insufficient as the disease progresses and patients show complications with vertebral collapse and severe deformity, which may lead to lumbosacral malunion and chronic lower back pain. To restore normal spinal alignment, prevent late‐onset neuronal damage, and allow early ambulation of patients, surgeons are increasingly adopting chemotherapy with surgery to solve this dilemma^6^.

Surgery promotes the quiescence of the abscessed area by lesion clearance, bone grafting, and internal fixation, so that the infection can be clinically eliminated^7^. However, as the transition zone is between the lumbar vertebrae and the sacrum, the unique anatomical structure and biomechanical characteristics of the lumbosacral segment bring challenges to the operation. The adjacent organs in the lumbosacral area not only need to be protected from damage, but one also needs to pay special attention to the reconstruction of lumbosacral stability after lesion removal^8^. Thus far, various surgical methods including an anterior approach, a combined anteroposterior approach and a posterior approach have been described for treating lumbosacral junction TB^9^, ^10^, ^11^. Albeit direct exposure of the lesion and removal of the damaged vertebrae and disc have the above advantages, the anterior approach is still too invasive and involves abdominal organs and iliac vessels as described by some scholars. Combined posterior and anterior approach is often related to prolonged operating time, large amount of bleeding, and increasing complications, which is unsuitable for TB patients with poor health conditions. Due to a lack of evidence and guidelines regarding optimal treatment and management strategies, the treatment of lumbosacral junction TB remains a difficult and challenging decision‐making process. Currently, reports on the management of lumbosacral junction TB are relatively scarce due to its low incidence rate.

Some surgeons reported the posterior approach can establish an effective operation channel and sufficient manipulation space by resecting partial posterior column structure of the infected vertebrae, so that the operation of the vertebral body 360° range can be achieved under direct vision on the outside of the dural sac. Such an approach can remove the majority of TB lesions and perform nerve decompression without damaging the spinal cord. However, for patients with severe bone destruction of the lumbosacral region, the posterior pedicle screw system was difficult to obtain sufficient bony structural support, resulting in failure to reconstruct the damaged spinopelvic complex. For patients whose S_1_ vertebral body can be implanted with pedicle screws, avoiding spinopelvic fixation can preserve more motion units. Therefore, the surgical treatment of TB in the lumbosacral junction is still a challenge for spinal surgery.

In this study, 38 patients with lumbosacral junction TB who underwent one‐stage posterior lumbosacral fixation or lumbopelvic fixation and postural drainage were followed up for more than 5 years. The aims of this research are: (i) to elucidate the clinical features and introduce a surgical procedure of lumbosacral junction TB; (ii) to evaluate the feasibility and safety of posterior approach in the treatment of lumbosacral junction TB; and (iii) to assess the medium‐term clinical outcomes and effectiveness of this surgical procedure in eliminating the infection, regaining lumbosacral or lumbopelvic stability, resolving neurological impairments, and restoring normal quality of life.

## Methods

### General Data

A total of 79 patients were diagnosed with lumbosacral junction TB at our hospital from February 2008 to July 2015. Of these, 38 patients received posterior approach. Those who did not receive posterior approach were treated with other procedures or anti‐TB drugs until their symptoms improved.

The inclusion criteria were as follows: (i) patients were diagnosed with lumbosacral junction TB, and all of them were adults; (ii) patients received surgical treatment due to presenting with persistent lower back pain caused by instability, severe or progressive neurological impairment, unpreventable progressive deformity, or weak efficacy of anti‐TB drugs, as confirmed by drug sensitivity testing; (iii) patients underwent one‐stage posterior debridement, interbody fusion, lumbosacral or lumbopelvic fixation, and postural drainage; (iv) complete follow‐up medical records more than 5 years.

The exclusion criteria were individuals who had: (i) already undergone lumbosacral surgery and thus showed unclear morphology in the area; (ii) had history of congenital scoliosis or ankylosing spondylitis; (iii) showed multi‐level lumbosacral junction TB (exceeding two segments that required surgical treatment) with iliac fossa or anterior abscess formation; or (iv) patients who have surgical contraindications after preoperative evaluation.

Written informed consent was obtained from all selected patients prior to the study, and the ethics committee of Xiangya hospital approved the study protocol. The primary diagnosis of tuberculous spondylodiscitis or spondylitis was made on the basis of clinical, neurological, hematologic, imaging, and laboratory data. Final confirmation of diagnosis was achieved through histopathologic analysis of a biopsied sample or a positive tubercle bacillus culture. The 38 patients included in the study were all the patients who were treated following the same protocol of our hospital for lumbosacral TB, as their conditions indicated. No patients were lost to follow‐up.

### Preoperative Procedure

Anti‐TB treatment with the HREZ chemotherapy regimen was routinely carried out for at least 2 weeks prior to surgery. The dosages of each drug were the following: isoniazid (H; 5 mg/kg/day, <300 mg/day), rifampicin (R; 10 mg/kg/day, <1200 mg/day), ethambutol (E; 15 mg/kg/day, <2500 mg/day), and pyrazinamide (Z; 30 mg/kg/day, <2000 mg/day). Nutritional support therapy was administered to correct hypoproteinemia and anemia. Surgery was postponed until erythrocyte sedimentation rate (ESR) values, which indicated the activity level of the TB infection, either decreased significantly or returned to normal. If a patient experienced cauda equina syndrome or dramatic aggravation of neurological impairment during the anti‐TB treatment, emergency surgery was performed regardless of the ESR value.

### Surgical Methods

#### Anesthesia and Position

The patients were placed in a prone position under general endotracheal anesthesia.

#### Exposure and Fixation

A posterior midline incision with length of 15 to 20 cm was made with the affected vertebral body as the center, the spinous processes, bilateral lamina, facet joints, and transverse processes were exposed. With the help of C‐arm fluoroscopy, pedicle screws were mounted on one or two segments above the infected vertebra. Pedicle screws were inserted in the S_1_ vertebra in cases where the vertebral body was slightly damaged so that the integrity of the pedicles was preserved. If the S_1_ vertebra and pedicle channel were severely damaged, iliac screws were fixed to the iliac wings.

#### Debridement and Nerve Decompression

**Fig. 1:**
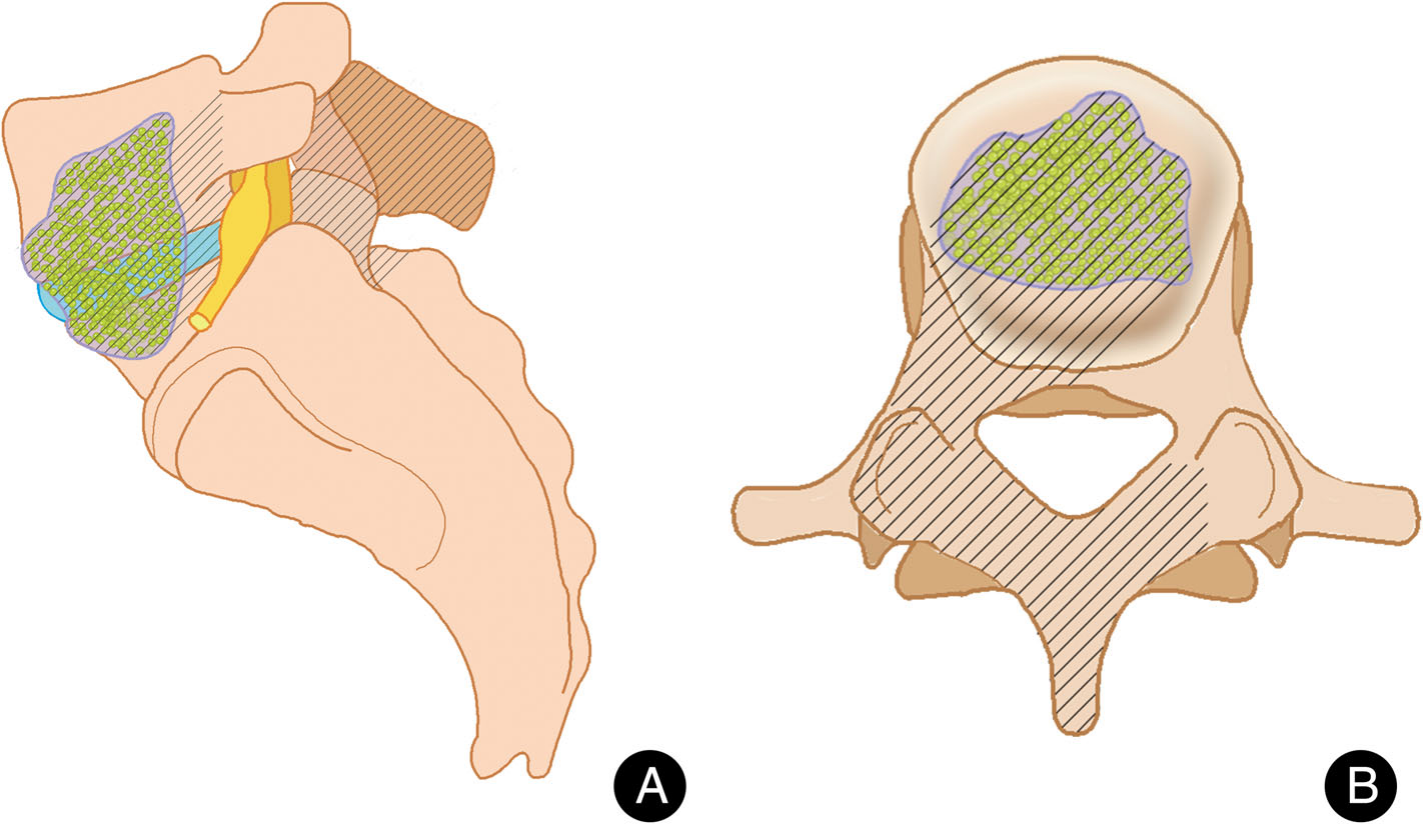
A posterior approach for lumbosacral junction TB. Black stripe area showed the range of excision including spinous process and unilateral (or bilateral) facet joint form both sagittal (A) and transverse plane (B).

A hemi‐laminectomy or laminectomy was performed on the severely damaged side of the lesion segment. The superior and inferior articular processes of the pathologic vertebrae were partially resected to expose the affected vertebral bodies. Curettes of varying angles were used to remove the lesion, including sequestrum, necrotic intervertebral disc, and tuberculous granuloma. Pus and necrotic tissue were removed by negative pressure irrigation and negative pressure suction *via* a soft catheter, which was placed deep into the lesion. The same procedure was performed on the other side, if necessary (Fig. 1).

#### Installation Rods and Bone Graft

Installation of permanently contoured rods and compression with a cantilever bending technique under vision were employed to achieve local spinal alignment. The bone surfaces of the vertebral body were scraped till bleeding and repaired as implant bed. A suitable allogeneic bone block was implanted between the vertebrae to complete the reconstruction of the anterior middle column. Moreover, autologous and allogeneic particulate bone was implanted between the bilateral transverse processes. Streptomycin (1 g) combined with isoniazid (0.3 g) were used locally in the focal area, and the incision was closed in layers after a drainage tube was placed.

Histopathologic examinations and *Mycobacterium* cultures were performed on the surgical specimens of each patient.

The operation was performed by the same group of surgeons. The surgical equipment was Medtronic CD HORIZON SOLERA Spinal Fixation System (USA) and Shanxi Aorui Biomaterials Co., Ltd. allograft bone block (China).

### Postoperative Procedure

Typically, the drainage tube was removed once the volume of drainage became less than 30 mL within 24 h. All patients continued with the HREZ chemotherapy regimen for 12–18 months after surgery. Intravenous antibiotics and nutritional support were administered to prevent infection. Rehabilitation training and physical therapy were provided to all patients as early as possible to help them regain neurological function and prevent thrombosis. With the assistance of a rigid lumbosacral brace, the patients were permitted to walk gradually after strict bed rest for 4 weeks post‐surgery. The external brace was removed when imaging data from the patients revealed formation of a callus. Routine blood and hepatorenal function tests, as well as ESR tests, were performed to monitor the side effects and to evaluate the clinical efficacy of the treatment. Regular re‐examinations through outpatient services were conducted every 3 months in the first year postoperatively, and every 6 months thereafter. At least 5 years of follow‐up was carried out for each patient to obtain clinical and radiologic data.

### Outcome Measures

The patient demographic, intraoperative bleeding amount and operative time were recorded. ESR was measured to indicate the activity level of the TB infection preoperatively and postoperatively. The visual analog scale (VAS) score was used to assess pain intensity. The Japanese Orthopaedic Association (JOA) score and Oswestry Disability Index (ODI) were used to evaluate neurological function and quality of life in patients, respectively. VAS, JOA, and ODI together with lumbosacral angle were measured preoperatively, postoperatively, and at the last follow‐up. The Kirkaldy‐Willis criteria was used to determine the functional outcome at the end of the observation period for each patient. Imaging approaches including radiography, computed tomography (CT), and magnetic resonance imaging (MRI) were used to detect narrowing of the intervertebral space, vertebral body collapse, spinal instability, bone destruction, and paravertebral and epidural abscess formation during diagnosis and preoperatively. Routine radiography and CT scans were also carried out postoperatively and at follow‐up to assess the placement of instruments and extent of decompression, as well as to classify bone fusion.

#### Demographic Data

The demographic data, including age and gender of the 38 patients, were recorded.

#### Blood Loss and Operative Time

The blood loss and operative time were recorded from anesthesia to incision closure.

#### Inflammation Indicators

Preoperative, postoperative, and last follow‐up ESR were recorded to comment on TB activity.

#### Visual Analog Scale

The VAS score was used for pain assessment. To be specific, a horizontal line on the paper divided into 10 equal portions was provided to the patient. The two ends represented 0 and 10 points, with 0 points meaning no pain and 10 points indicating unbearable pain. The patient then drew a mark on the horizontal line to indicate the degree of pain according to the patient's own feelings with 2–4 points for mild pain, 5–7 points for moderate pain, and 8–9 points for severe pain.

#### Japanese Orthopaedic Association

The JOA score consists of four sections (subjective symptoms, clinical signs, activity restrictions, urinary bladder function) quantifying functionality and pain. The detailed methods were reported in a previous study, with lower scores indicating poorer conditions^12^.

#### Oswestry Disability Index

The ODI measures the impact of low back pain on the patients' functional ability in 10 domains of activities in daily life, including pain intensity, personal care, lifting, walking, sitting, standing, sleeping, sex life, social life, and traveling. Previous literature reported the assessment methods, with higher score indicating higher disability^13^.

#### Kirkaldy‐Willis Criteria

Kirkaldy‐Willis *et al*.^14^ reported the modified criteria for functional outcome, which included four categories: excellent, good, fair, poor.

#### Lumbosacral Angle

The lumbosacral angle was used to assess deformity correction, which was measured as the angle of intersection between the vertical lines of the L_5_ upper and the S_1_ upper margins.

#### Bone Fusion

The bone fusion status was evaluated according to the modified radiologic criteria by Lee *et al*.^15^.

### Statistical Analysis

Statistical analyses were performed using SPSS version 20.0 (IBM Corp., Armonk, NY). The continuous variables including ESR, VAS score, JOA score, ODI, and lumbosacral angle were collected preoperatively, postoperatively, and at the final follow‐up. The paired *t*‐test was applied on the data (preoperative *vs* postoperative and at the final follow‐up). Discrepancies of the normal distribution were evaluated using the rank sum test. The data was expressed as mean ± standard deviation. A *P* value < 0.05 was considered statistically significant.

## Results

### General Results

The patient demographic data and disease characteristics are detailed in Table 1. Among the patients who met the inclusion criteria, 24 were male and 14 were female. The mean age at time of surgery was 48.0 ± 13.0 years (range, 25–75 years). All cases presented with TB symptoms such as low back pain, weakness, weight loss, and low fever. In addition, 18 cases (47.4%) had radicular pain, nine cases (23.7%) showed lower limb dysfunction, and one patient (2.6%) suffered from cauda equina syndrome. The ESR values were found to be raised at the initial stage before surgery. Histopathologic examinations of surgical specimens from all 38 patients confirmed the presence of tuberculous granuloma or caseous necrosis. Of these, 11 cases showed positive culture for *Mycobacterium tuberculosis*. The average length of the postoperative follow‐up was 66.2 ± 4.4 months (range, 60–78 months). All patients were cured and had no recurrence of TB.

**TABLE 1:** Clinical evaluation indexes for preoperative, postoperative, and final follow‐up (mean ± standard deviation)

Index	Preoperative	3 months postoperative	Final follow‐up
ESR (mm/h)	67.9 ± 21.9	11.8 ± 2.6^*^	7.6 ± 2.1^*^
JOA	18.5 ± 2.9	23.2 ± 1.9^*^	26.9 ± 1.1^*^
VAS	7.2 ± 0.9	2.9 ± 0.8^*^	0.8 ± 0.7^*^
ODI	44.3 ± 6.7	22.9 ± 5.1^*^	9.3 ± 1.9^*^

JOA, Japanese Orthopaedic Association; ODI, Oswestry Disability Index; VAS, visual analog scale.

^*^ *P* < 0.01.

### Blood Loss and Operative Time

The average intraoperative bleeding amount was 726.3 ± 151.9 mL (range, 400–1100 mL), and the average operative time was 137.6 ± 22.5 min (range, 110–200 min).

### Inflammation Indicators

The ESR values were 67.9 ± 21.9 mm/h preoperatively, which significantly decreased to 11.8 ± 2.6 mm/h at 3 months post‐surgery (*P* < 0.01) and 7.6 ± 2.1 mm/h at the final follow‐up (*P* < 0.01).

### Constant Functional Score

#### Visual Analog Scale

The VAS score significantly decreased from an average of 6.8 ± 1.1 preoperatively to 2.9 ± 0.8 at 3 months postoperative (*P* < 0.01). The value continued to decline to 0.8 ± 0.7 at final follow‐up (*P* < 0.01, compared to preoperative).

#### Japanese Orthopaedic Association

Scores for neurological function (JOA) at 3 months postoperative showed significant improvement when compared with preoperative scores (23.2 ± 1.9 *vs* 18.5 ± 2.9) (*P* < 0.01). At the last visit, the score increased markedly to 26.9 ± 1.1 (*P* < 0.01, compared to preoperative).

#### Oswestry Disability Index

The mean ODI score before surgery was 44.3 ± 6.7, while the mean ODI score was 22.9 ± 5.1 and 9.3 ± 1.9 at 3 months postoperative and the final follow‐up, respectively. There were statistical differences between pre‐operation and 3 months postoperative and final follow‐up (*P* < 0.01, compared to preoperative).

#### Kirkaldy‐Willis Criteria

At the end of the follow‐up period, patients' functional outcome was assessed using the Kirkaldy‐Willis criteria; 21 cases reported excellent results, 16 cases reported good results, and one case reported fair result. There were no reports of poor outcomes.

### Lumbosacral Angle

The lumbosacral angle significantly increased from the preoperative average of 21.7° ± 1.8° to an average of 26.4° ± 1.4°, postoperatively (*P* < 0.01), with the correction rate of 22.3% ± 6.2%. At the last visit, the lumbosacral angle was an average of 25.3° ± 1.3° (compared to preoperatively, *P* < 0.01), with a correction loss of only 1.2° ± 0.7°. There was no statistically significant difference between the lumbosacral angle measurements made immediately post‐surgery and at the last follow‐up (*P* > 0.05).

### Fusion Time

**Fig. 2:**
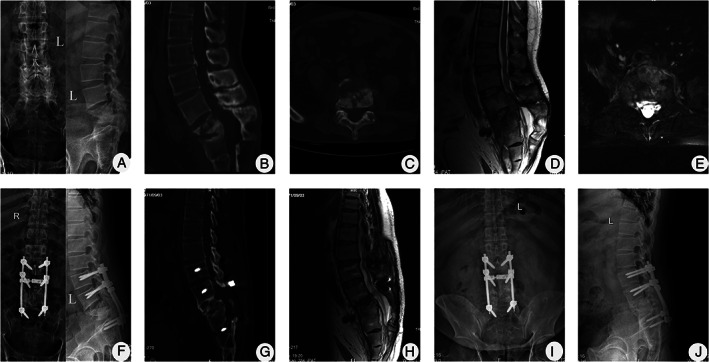
A 39‐year‐old male with L_5_/S_1_ TB underwent one‐stage posterior debridement, interbody fusion, and lumbosacral fixation. (A) Anterior and lateral radiographs of the lumbar spine showing the affected vertebrae with a lumbosacral angle of 22°. (B–E) Preoperative CT and MRI demonstrating that the L_5_ vertebral body was severely damaged and collapsed, with cold abscess formation in the spinal canal. (F) Postoperative X‐ray showing good internal fixation position with a lumbosacral angle of 28°. (G, H) Postoperative CT and MRI showing satisfactory bone fusion at 15 months and an unobstructed spinal canal. (I, J) X‐ray displaying solid bone fusion and no displacement of instruments, with the correction loss of 1° throughout 63 months of follow‐up.

**Fig. 3:**
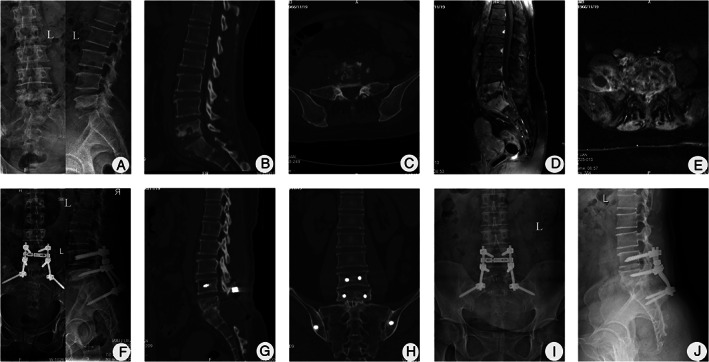
A 52‐year‐old female with L_5_/S_1_ TB underwent one‐stage posterior debridement, interbody fusion, and lumbopelvic fixation. (A–E) Preoperative images showing that the TB lesion was located at L_5_/S_1_ and the lesion involved vertebral body destruction (the pedicle of S_1_ was also affected) and abscess formation. The lumbosacral angle was 23°. (F) Postoperative radiograph depicting correction of the deformity and the lumbosacral angle at 28°. (G, H) CT showing solid bone fusion was achieved at 15 months. (I, J) X‐ray displaying the instruments in the correct position, with a lumbosacral angle of 27° at the follow‐up period of 60 months.

**Fig. 4:**
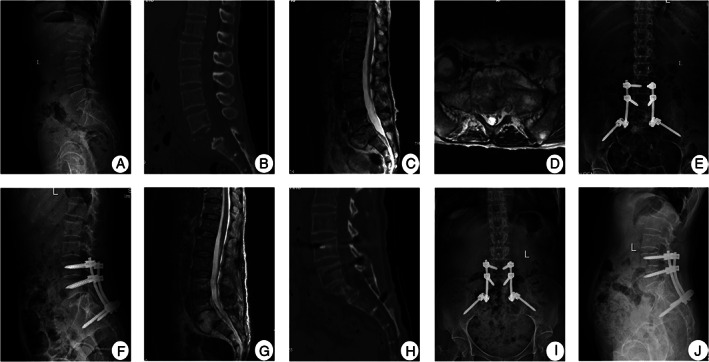
A 64‐year‐old female with L_5_/S_1_ TB underwent one‐stage posterior debridement, interbody fusion, and lumbopelvic fixation. (A–D) Preoperative images showing that the TB lesion was located at L_5_/S_1_ and the S_1_ vertebra was severely affected and collapsed, with prevertebral cold abscess formation. The lumbosacral angle was 24°. (E, F) Postoperative anterior and lateral radiographs of the lumbar spine demonstrating good position of posterior screw rod system, and lumbosacral angle was 27°. (G) Postoperative MRI showing most of abscess was eliminated. (H) CT showing bone fusion was obtained at 9 months. (I, J) X‐ray displaying good position of internal fixation, with a lumbosacral angle of 26° at the final follow‐up period of 72 months.

Spontaneous intervertebral bone fusion was achieved at 12.8 ± 1.9 months (range, 9–15 months) after surgery. Nonunion, pseudoarthrosis, loosening, or fracture of instruments had not occurred at the time of the final follow‐up (Figs 2‐4).

### Complications

None of the patients died or suffered permanent neurological damage. Two cases experienced superficial incisional infection, which was successfully controlled with antibiotics. Anti‐TB drug‐induced liver dysfunction occurred in a single patient and was subsequently managed using modified chemotherapy combined with hepatic protection treatment.

## Discussion

### Characteristics of Lumbosacral Junction TB

The lumbosacral spine is at the junction of a lordotic motion segment and a kyphotic fixed end, an area where the stress of the entire torso is concentrated. The intact facet joints and intervertebral discs of the lumbosacral segment are the main structures that resist the shear force of the L_5_ vertebral body forward. Hence, spondylolisthesis of the lumbosacral region can easily bring about force imbalance, such that firm internal fixations are usually required. Lumbosacral TB is relatively rare, and has an insidious onset and atypical symptoms. Individuals with the condition often report nonspecific symptoms, which are easily mistaken for spinal degenerative disease at the initial stage^16^. As the disease progresses, *M. tuberculosis* erodes the vertebrae to the point of sequestrum and abscess formation, leading to spinal instability or deformity. Additionally, nerve compression from TB lesions that invades the spinal canal potentially cause radicular pain, and even acute paralysis, which has deleterious effects on the patient's overall quality of life^17^.

### Therapeutic Method of Lumbosacral Junction TB

Standard systemic anti‐TB chemotherapy remains the basic strategy throughout the entire treatment period^18^, ^19^. In our study, normative anti‐TB treatments were carried out on all patients for 2 weeks preoperatively and for 12–18 months postoperatively. Common indications for surgery in lumbosacral junction TB are ineffective conservative measures, persistent pain unrelieved by chemotherapy, progressive neurological deficits, relatively massive cold abscesses, and extensive vertebrae destruction with instability or deformity. The surgery is specifically carried out with the goal of removing the lesion, relieving nerve compression, and rebuilding spinal stability, all of which aid the maintenance of normal lumbar lordosis and sacral kyphosis. This also ensures that the normal physiological line of force transduction is preserved. For patients who require surgery, the key techniques for surgical treatment of spinal TB include complete debridement, adequate bone grafting, and firm internal fixation.

### Surgical Approaches for Lumbosacral Junction TB

The surgical method for the treatment of TB in the lumbosacral junction is still controversial^20^, ^21^, ^22^. An anterior approach has been conventionally preferred since most TB lesions involve the vertebral body and intervertebral discs. The anterior approach allows for direct access where thorough debridement of the affected tissue and intervertebral reconstruction can be performed^23^, ^24^. Nevertheless, the anterior approach is associated with anteriorly related complications. In particular, the lumbosacral region is an area with complex local anatomical structures and is adjacent to numerous vital organs, including major blood vessels, nerve plexus, and ureters^22^. Tribus *et al*.^25^ reported that the distance from the bifurcation of iliac vessels to the top of the L_5_‐S_1_ disc averaged 18 mm, and the average space available between the left common iliac vein and the right common iliac artery is 33.5 mm, a result they surmised from studying 37 human cadavers. This means that it is too risky to perform the surgery *via* the anterior approach, due to the close proximity of the vasculatures that this approach exposes. In addition, anterior instruments may be inadequate since the presence of the TB infection is associated with osteoporosis that renders the vertebrae increasingly fragile, thereby potentially causing graft failure and loss of correction after surgery^26^. These existing objective factors pose significant challenges to anterior fixation of the lumbosacral segment, while carrying the risk of damage to major neurovascular structures. Some surgeons report that anterior debridement combined with posterior instrumentation in the management of lumbosacral TB can obtain favorable clinical efficacy^27^, ^28^. This approach overcomes the stability‐related shortcoming when the anterior approach is used alone and encompasses a broader surgical vision and higher bone graft fusion rate. However, it also results in superimposed surgical trauma and increased complications. Taking the above factors into account, we favor the posterior approach for treating lumbosacral junction TB when surgery is indicated.

### Feasibility and Safety of Posterior Approach

With the widespread application of pedicle screw‐rod internal fixation systems, the satisfactory safety and clinical efficacy of the posterior‐only approach for the treatment of lumbosacral TB has increasingly attracted the attention of orthopaedic surgeons for many reasons^7^, ^17^, ^26^, ^29^, ^30^. First, comparing the complex anatomical structures of anterior approach, the posterior approach is less invasive and avoids damage to vital nerves and blood vessels, allowing early rehabilitation training of the patients. Second, pedicle screws provide better holding force, and are thus superior to the anterior approach in correcting severe kyphosis. Third, this approach provides adequate exposure of the anterior part of the spinal canal, and allows posterior decompression, unilateral anterior decompression, rendering good clinical result. Fourth, the posterior internal fixation keep the instrumentation away from the tuberculous focus, conducive to the healing of TB. Zeng *et al*.^7^ successfully treated 15 cases of lumbosacral TB with paraspinal abscess using the posterior approach. Similarly, Xu *et al*.^17^ also reported that kyphotic deformity significantly improved with complete recovery of neurological function by interbody fusion and posterior lumbopelvic fixation for lumbosacral TB.

The posterior approach treatment of lumbosacral TB should follow an individualized protocol. A routine CT scan and MRI examination are essential before surgery to map out the details of the lesion. When the S_1_ vertebral body is severely damaged, the use of sacral pedicle screws cannot obtain sufficient bony support. The physiological force transmission line in the lumbosacral region is subsequently affected, which brings challenges to surgical reconstruction. The sacrum forms the posterior wall of the pelvis and is connected to the ilium through the sacroiliac joints. Physiologically, gravitational force is transmitted from the lumbosacral joint to the upper part of the sacrum, and then to the pelvis through the bilateral sacroiliac joints. Due to the special role it plays in pressure bearing and force transduction, the sacrum is considered the keystone of the pelvic ring^31^. For cases when S_1_ is severely damaged, lumbopelvic fixation can effectively relieve the stress on the diseased vertebra and promote its healing. In addition, iliac screw fixation can immediately stabilize the lesion and restore the normal lumbosacral angle^32^. For patients with an S_1_ vertebral body lesion that is not serious and with an intact pedicle channel, sacral pedicle screw fixation can effectively reconstruct local stability and cross spinopelvic fixation can be avoided.

### Medium‐Term Clinical Outcomes and Effectiveness of Posterior Approach

*M. tuberculosis* tends to infect the anterior and middle column of the spine. The controversy with regard to surgical treatment of lumbosacral TB with the posterior approach mainly centers around whether this approach can allow for complete debridement given its limited field of view. Indeed, the posterior approach offers no advantage in lesion clearance. Nonetheless, resection of both sides of the lamina and facet joints, and moderate stretching of the dura mater and nerve roots can provide enough operating space for the removal of the lesion. Then, through intraoperative pressure washing, negative pressure suction, and postoperative postural drainage, the sequestrum, necrotic tissue, and most abscesses can be removed. The remaining small areas of infection can be resolved by standardized anti‐TB chemotherapy after surgery^33^. Indeed, following the above techniques and procedures, all patients in our study achieved clinical cure without TB recurrence or intraspinal TB infection. Some surgeons are concerned that this surgical method destroys the posterior column and may cause spinal instability. However, pedicle screws, which allow for three‐column fixation, can effectively restore the normal physiological curvature of the spine, and overcome the instability caused by column damage within a short time period. Sufficient intervertebral and transverse bone grafting provide long‐term bone support for spine stability^34^. In this study, during the medium‐term follow‐up over 5 years of this study, there were no cases of TB recurrence from incomplete lesion removal, all 38 cases showed complete resolution from TB infection by the last follow‐up. The patients who suffered preoperative neurological deficit had satisfactory recovery at the final follow‐up. In terms of spinal stability, over the 5 years of the observation period, patients presented an average loss of the correction angle of only 1.2° ± 0.7° with a 22.3% ± 6.2% correction rate after surgery. Pain intensity as measured by VAS dropped by over 80%. In terms of structural recovery, all patients achieved satisfactory bone fusion at an average of 12.8 ± 1.9 months after surgery without a single incidence of instrument failure. While at the last follow‐up, 21 patients (55.3%) reported excellent status, and 16 patients (42.1%) reported good status based on Kirkaldy‐Willis criteria.

### Limitations

This study has some limitations. First, this is a retrospective study, which may lead to biased results. Furthermore, this study is a single‐center study with a relatively small sample size. Prospective studies with larger sample sizes are required to confirm the findings, although given the rarity of this condition, it may take considerable time and coordination for such studies to take place.

### Conclusion

For patients with lumbosacral junction TB where anti‐TB chemotherapy is ineffective, or with worsening neurological deficits and pain, one‐stage posterior debridement, interbody fusion, lumbosacral or lumbopelvic fixation, and postural drainage, according to the severity of sacral destruction, is an effective and highly safe procedure. This surgical approach can lead to reconstruction of lumbosacral or lumbopelvic stability, while facilitating healing from TB‐related inflammation.

#### Authorship Declaration

All authors declare no competing interests.
